# The effectiveness and cost effectiveness of the PAtient-Centred Team (PACT) model: study protocol of a prospective matched control before-and-after study

**DOI:** 10.1186/s12877-015-0133-x

**Published:** 2015-10-23

**Authors:** Trine S. Bergmo, Gro K. Berntsen, Monika Dalbakk, Markus Rumpsfeld

**Affiliations:** Norwegian Centre for Integrated Care and Telemedicine, University Hospital of North Norway, 9038 Tromsø, Norway; Division of Internal Medicine, University Hospital of North Norway, Tromsø, Norway; Department of Clinical Medicine, University of Tromsø - The Arctic University of Norway, Tromsø, Norway

**Keywords:** Interdisciplinary teams, Patient centeredness, The chronic care model, Integrated care, Frailty, Multiple conditions, Health service research, Matched case-control study, Health outcomes, Health care costs and cost-effectiveness analysis

## Abstract

**Background:**

The present study protocol describes the evaluation of a comprehensive integrated care model implemented at two hospital sites at the University Hospital of North Norway (UNN). The PAtient Centred Team (PACT) model includes proactive, patient-centred interdisciplinary teams that aim to improve the continuum and quality of care of frail elderly patients and reduce health care costs. The main objectives of the evaluation are to analyse the effectiveness and cost effectiveness of using patient-centred teams as part of routine service provision for this patient group. The evaluation will analyse the effect on patient health and functional status, patient experiences and hospital utilisation, and it will conduct an economic evaluation. This paper describes the PACT model and the rationale for and design of the planned effectiveness and cost-effectiveness study.

**Methods/design:**

This is a prospective, non-randomised matched control before-and-after intervention study. Patients in the intervention group will be recruited from the hospital sites that have implemented the PACT model. The controls will be recruited from two hospitals without the model. The control patients and the index patients will be matched according to sex, age and number of long-term conditions. The study aims to include 600 patients in each group, which will provide sufficient power to detect a clinical change in the primary outcome. The primary outcome is the physical dimension of the Short Form Health Survey (SF-36). Secondary outcomes are the Patient Generated Index (PGI), the Patient Activation Measure (PAM), the Patient Assessment of Chronic Illness Care (PACIC), hospitalisation and length of stay. The cost-effectiveness study takes a health provider perspective and calculates the cost per quality-adjusted life-years (QALYs) gained. The data will be collected at baseline, 6 and 12 months. The data will be analysed using techniques and models that recognise the lack of randomisation and the correlation of cost and effect data.

**Discussion:**

The study results will provide knowledge about whether the integrated care model implemented at UNN improves the quality of care for the frail elderly with multiple conditions. The study will establish whether the PAC. T model improves health and functional status and is cost effective compared to the usual care for this patient group.

**Trial registration:**

ClinicalTrials.gov: NCT02541474

## Background

As the population ages, the proportion of older persons admitted to hospital is continuously increasing. Older people are found to have higher admission rates and longer hospital stays than the general population [1]. Patients aged 67 years and older account for 35 % of the hospital admissions in Norway [2]. This is comparable to other countries. Persons aged 65 and over account for approximately 38 % of the hospital admissions in the United Kingdom [1] and 36 % in the United States [3]. Older people are also major consumers of hospital-based acute services [4]. The current disease-oriented and episodic models of care do not adequately respond to the complex care needs of older patients [5] and patients with multimorbidity [6]. Compared with younger persons, older adults have longer stays, their visits have a greater level of urgency, they are more likely to be readmitted, and they experience higher rates of adverse health outcomes after discharge [5]. Studies show that there is a considerable risk of adverse events in relation to the transition of patients between hospitals and primary care services and that information exchange at discharge is often insufficient [7, 8]. Thus, there is a growing need for better care coordination between and within primary and specialist health care services to ensure patient safety and continuity of care [9].

The efficient and effective management of acutely ill, complex inpatients poses one of the greatest challenges in current hospital care [10]. Health care utilisation and costs increase significantly with increasing number of chronic conditions [11]. One way to improve the management of the older patients with multimorbidity is to use patient-centred interdisciplinary teams. Interdisciplinary team work offers an integrated approach to providing coordinated health care to “high-risk” patients with complex long-term needs. Team work has been considered good practice for more than two decades [12]. However, little evidence exists that these types of interventions are effective. Five recent systematic reviews found mixed results and they reported that there exists no clear evidence that these interventions are effective or cost effective [4, 13–16]. These reviews reported that some well-integrated models have improved the process of care and have the potential to reduce hospitalisation and nursing-home use [13]. Some of the studies also reported improved prescribing and medication adherence [14]. One of the reviews that specifically looked into the cost effectiveness of multidisciplinary teams found the same trend of insufficient evidence. This review reported mixed results and a high degree of heterogeneity and low-quality evaluations [15]. All these reviews concluded that more research is needed in this area. They also argued that comparison among studies is difficult because of the heterogeneity in both how interdisciplinary teams work and the study populations analysed. This hampers the ability to develop best-practice models.

In our local setting, a recent report documented that two of the main challenges are the lack of attention to the personal context of the patients and the fragmented care delivery [17]. The need for a re-design of care delivery with more service integration and improved follow-up regimen seems particularly urgent for frail elderly patients with complex and long-term needs [18–20]. A large-scale project that facilitates more integrated and coordinated care for frail elderly medical patients is now under development at the University Hospital of North Norway (UNN). The Patient-Centred Team (PACT) model is being established at UNN Tromsø, UNN Harstad and their hosting municipalities. The purpose of the PACT model is to improve the continuum and quality of care for frail elderly patients and to reduce health care costs. The PACT model has been initiated and is solely driven by managers and clinical personnel at the hospital and at the community services.

The PACT model is inspired by the Chronic Care Model (CCM) and builds on two pillars. These are “the informed active patient” and “the pro-active prepared health care team”, engaging in “productive interactions” for “health and functional outcomes”. Both health management’s support and use of information and communication technology (ICT) are key supporting factors [21]. There is a growing evidence base for CCM’s effects on both care processes, health outcomes [22, 23] and cost savings [24]. CCM continues to inspire care reforms internationally [25, 26]. The Norwegian health authorities are pushing for a large system transformation towards a truly person-oriented integrated care model which broadly aligns with CCM-principles [18].

The present paper describes the effectiveness- and cost-effectiveness evaluation of the PACT model. The main aim of the evaluation project is to determine whether the PACT model improves health and functional status and is cost effective compared to usual care. First, we describe the PACT model now being implemented as part of routine care at two hospitals in northern Norway and their hosting municipalities. Second, an overview of the design and methods used in the effectiveness study and the economic evaluation is provided, followed by a brief discussion. The evaluation project is funded by a research grant from the regional health authorities Helse Nord (Grant No. HST1242/3-15).

### The PACT model

The PACT model is a comprehensive integrated care model that aims to ensure safe discharge and prevent hospital admissions for frail elderly patients with multiple conditions. The patient-centred and proactive interdisciplinary teams, which include both the community services and hospital staff, are the key element of the model. The main task of the team is to follow patients through the system and help them receive appropriate and timely health care services when needed at the most appropriate location. The team will include the patients and their families in planning and facilitating service integration. The patients will be actively involved in defining goals, assessing needs and making care plans and follow-up protocols. Patient involvement and engagement in care has been shown to improve health and functional outcomes [27]. The team will identify and assess needs early, provide support during discharge and follow-up, facilitate coordination and integrated services across the levels of care and support, and care for the patients outside the hospital. This might ease the transition, reduce unplanned hospital admissions, reduce the need for community services and improve or avoid deterioration in patient health outcomes.

The PACT model includes five main tasks: to identify the patients with a special need for coordinated care; to identify patient goals and conduct need assessments; to facilitate individualised care plans and tailored follow-up protocols; and to initiate meetings regarding the coordination and integration of patient care across service providers. In particular, the team will assess the risk of fall and the need for special aid and review medication lists. The team will also ensure that general practitioners (GPs) are informed and included in the planning process. The patients will remain a team responsibility until the community service provider has established appropriate care or until the patients can manage at home without help.

The core team consists of the team leader (ICL) and two full-time coordinators: one from the hospital and one from the community service. The core team will be responsible for the day-to-day management of the teamwork and ensuring that the patients receive appropriate and timely support and services. The interdisciplinary team further consists of a senior medical doctor, geriatric nurses, district nurses, physiotherapists, occupational therapists and one pharmacologist.

The local implementation and structure of the PACT model will vary between the two experimental regions, even if the service model has a formal selection procedure and a common core of services. The structure and organisation of the service model will cover complex areas such as patient logistics (regulating the patient flow through the system; knowing when and how blockages occur and resolving them; using various instruments, such as case management; discharge protocols; capacity constraints; and home monitoring and support with various means). Information logistics, such as team communication and information exchange, will also vary. The general aim of the PACT model, however, will be the same: to ensure that the patients receive the right treatment and care at the right time and in the most suitable location.

#### Status

At the time of writing, all team members, including a part-time recording secretary, have been appointed at the hospital in Tromsø. The test and developing phase is well underway. The team is now actively promoting their existence and services by visiting GPs, district nurses, other community service providers and hospital wards and clinics. The first patients have been referred to the team as part of the test phase, but no patients have yet been enrolled in the trial. The team has been assigned office space at the hospital and has scheduled two daily meetings to plan activities and discuss patients. In between team meetings, the team is conducting home visits, attending coordination meetings, writing case reports and updating patient records. The second hospital has finished the planning phase and will be operational by fall 2015.

## Methods/design

### Objectives and research questions

The main objective of the evaluation is to analyse the effectiveness and cost effectiveness of establishing patient-centred interdisciplinary teams (the PACT model) as part of routine health care delivery for frail elderly patients with multiple conditions. The evaluation will analyse the effect of the PACT model on patient health and functional status, patient experiences and hospital utilisation, and we will conduct an economic evaluation. The research questions (RQs) include the following:

#### Effectiveness evaluation

1. Does the PACT model affect the health outcomes and functional status of the patients?
2. Does the PACT model improve patient experiences and have an impact on the areas in life that the patients themselves consider important?
3. Does the PACT model reduce unplanned hospital admissions and length of stay?

#### Cost-effectiveness evaluation

4. Does the PACT model represent cost-effective resource use from a health provider perspective in the short term (trial-based evaluation)?
5. Is the PACT service model more cost effective for any specific subgroups of patients, defined by age, conditions (diagnoses), co-morbidities and other baseline factors?
6. Is the PACT model cost effective in a 10-year perspective (model-based evaluation)?

### Design

This evaluation study is a prospective non-randomized, matched control, before-and-after study [28]. We will compare all hospitalised patients meeting the inclusion criteria with patients receiving usual care in a control setting. The PACT model is directed at the organisational level or geographic site, affecting all patients within the uptake area. Therefore, the intervention and controls are separated in different hospitals to avoid contamination. The chosen design improves comparability between the patient populations by matching them on factors known to be important for outcomes (age, sex and number of conditions), adjusting for baseline differences between the populations with a before-after design and adjusting for known confounders by the propensity score method [29, 30].

### Participants

The intervention group participants will be recruited consecutively until we have reached the target of 600 patients. As soon as the patients have signed the consent form, the intervention will start and the baseline data collection will begin. The follow-up period will be one year from the initial assessment for each patient.

- Inclusion criteria: Patients with emergency admission to the UNN internal medicine department in Tromsø and Harstad who are over the age of 65 years, have two or more long-term conditions and provide valid written informed consent (either by the patient or the next of kin).
- Exclusion criteria: Language barriers, life expectancy of less than three months and unable to provide written informed consent.

The control population will be recruited from the Nordland Hospital in Bodø and from the hospital UNN Narvik. When an index patient is identified in the hospitals using patient-centred teams, at least two eligible patients from the control hospitals will be identified. Sex, age and number of long-term conditions will be used to match the control patients to the index patients. The control patients will be subject to the same data collection as the intervention patients.

### Sample size

The sample size calculation is based on the primary outcome measure: the Short Form Health Survey (SF-36). The SF-36 has a range from 0 to 100, where 100 is the best possible score, and a standard deviation (SD) of approximately 25 in other populations. Points of two have been shown to be clinically significant [31]. Two different geriatric integrated care interventions similar to ours found differences of 4–6 points between intervention and control groups [32, 33]. We wish to be able to detect a difference of four points in the SF-36 physical health dimension between the intervention and control groups. With 600 patients in each group and a statistical significance level of 0.05, we will have a power of 0.80 to detect such a difference between the groups. We expect this to be realistic based on the existing hospital admission rates. The intervention and control hospitals had approximately 9500 medical emergency admissions for patients aged 67 years and older in 2014 [2].

### Setting

Trials are often performed on selected patients without comorbidities, causing studies to suffer from limited external validity [34]. Van Royen et al. (2014) argue that there is a driving demand for real-word clinical practice data [35]. In this project, the team members will develop the PACT working routines as part of the daily activities at the hospitals. The model will include medical patients over 65 from an everyday clinical setting, making the trial context naturalistic. The real-world setting and normal patient caseload make the design resemble usual care, thus increasing the generalisability from the trial to other patients in regular practice.

### Outcome measures

The outcomes measures need to match the desired effects of the intervention: the triple aim of improved health and function, improved patient experience and lower costs. The primary outcome is the adjusted differences in the change in the physical health dimension of the Short Form-36 (SF-36) between the intervention and the control groups.

The secondary outcomes are the other dimensions of the SF-36 instrument, health resource use (to measure the costs both in primary and secondary care) and the patient-generated index (PGI) [36]. The PGI registers the patient’s own health complaints at baseline and the severity of these at baseline and follow-up points (the PGI-open format) [37]. The Patient Activation Measure [38] will also be used to measure health self-efficacy. We will use the Patient Assessment of Chronic Illness Care Questionnaire (PACIC) adapted for the Norwegian context [39] to measure how well the patients think the service has been able to support pro-active patient centred care.

Other variables that will be collected are self-reported background information on life-style, socio-economic status and previous health care experiences.

### Data collection and management

Each hospital will have a part-time study nurse based in the geriatric department. The study nurses will administer the consent forms and support the data collection from the patients. The background variables and all the questionnaires will be completed by the patients at baseline, 6 and 12 months.

All data on health service use, including medication, will be collected from electronic information systems. Data on hospital use (hospital admission, length of stay, outpatient consultation, emergencies and medication) will be collected from the hospital records. Data on primary health resource use will be collected from the GPs, the municipalities and other available information systems. Data from the municipalities will supplemented by interviews with the providers. The cost associated with the PACT model itself will be determined by interviewing the PACT members (staff) and reviewing budgets.

### Effectiveness evaluation

#### Health and functional status

To measure health and functional status, the SF-36 was chosen. This is an instrument which researchers across the world have used to describe health-related quality of life in over 4000 publications [40]. Its validity and reliability are high and well documented [41]. The questionnaire has eight domains: physical function, role-physical, bodily pain, general health, vitality, social functioning, role-emotional and mental health. Since the patients in this study will be recruited in a somatic-care setting, the physical function sub-domain has been chosen as the primary outcome measure. The other domains are secondary outcomes.

The scores of the SF-36 do not follow a normal distribution, yet the accepted convention among authors is to use means, standard deviations and ANOVA in statistical analyses. This is possible and desirable because of the high robustness of these statistical methods in the face of non-normal distributions, and because they allow comparison of results with previously published results [42].

#### Patient experiences

The CMM-theory assumes that patients who experience a person-centred approach are more likely to have their personal goals addressed. Furthermore, the CMM assumes that person centeredness improves self-efficacy for health issues and that the patients experience care and service provision that is more sensitive to their needs. The instruments we will use to assess patient experiences are listed below.

The PGI [36] will be used to measure the patients’ personal goal attainment. The PGI registers patients’ own health complaints and their severity at baseline and follow-up [37].

The Patient Health Activation (PAM) instrument will be used to measure self-efficacy. The concept of an informed active patient presumes that if we support a person in their management of their own condition, this will translate into better health and functioning. However, self-management is contingent on motivation, knowledge and skills [38].

The PACIC will be used to assess if the PACT model is in accordance with the pillars of the CCM. The PACIC is developed to measure the presence of patient engagement in self-management, patient involvement in the development of a care plan and the degree of individualised goal setting. The instrument also measures the presence of identifying and solving barriers during care plan implementation as well as the degree of progress of and revisions to the care plan [39]. The PACIC has been validated, shows good reliability and has been widely used. However, correlations between high PACIC scores and care outcomes vary [43–46]. Recent publications indicate that CCM-care is especially important among disadvantaged patients, such as minority groups and patients with a low health activation [44, 47]. In the absence of any other instrument for the purpose of measuring fidelity to CCM-care, we will use the PACIC questionnaire to determine experienced fidelity to the CCM and to explore the interaction with the results collected by the PAM instrument.

#### Service use

Hospital admission and length of stay will be used as intermediate outcome measures to assess how the PACT model affects hospital utilisation.

#### Analyses

The analyses will use an intention-to-treat approach that includes all participants who signed the informed consent at the intervention and control hospitals. Missing data will be imputed using multiple imputations [48]. The multiple imputation model will include predictors from all time points, including health-related quality of life (SF-36), background variables, trial-related variables and resource use at baseline. Results with and without imputed data will be reported. The data will be analysed as a synthetic randomised controlled trial (RCT), using the propensity score method. This method postulates that each patient has a basic probability of the outcome of interest based on his/her characteristics [29]. Based on available knowledge for each patient (e.g. age, sex, education, ethnicity), we can calculate the patient’s propensity for the outcome (i.e. change in QoL). In subsequent analyses, we stratify analyses by this score. Within each stratum, the only known difference between patients with respect to the outcome will be the patient-centred teams. The fundamental requirement for validity in a causal analysis is that the variables used to create the propensity score are independent of the probability of getting the intervention. In common-sense terms, this means that in the synthetic study, within the same propensity score strata, the assignment to treatment is essentially the same as if it had been done by randomisation.

### Cost-effectiveness evaluation

#### Trial-based economic evaluation

The trial-based economic evaluation consists of a cost-utility analysis with a time horizon of 12 months. The analysis takes a health provider perspective and collects data on health resource use and health outcomes measured in quality-adjusted life-years (QALYs). The economic evaluation will be conducted in accordance with the CHEERS statement [49].

For the economic evaluation, health outcomes will be measured using the SF-6D instrument [50]. The SF-6D will be extracted from the SF-36 and will include physical functioning, role participation (combined role-physical and role-emotional), social functioning, bodily pain, mental health and vitality. The algorithm by Brazier et al. will be used to transform the scores into utility values [51]. The SF-6D scores will be transformed to QALYs by weighing the per-period estimates of utility scores with the time spent in the particular health state, assuming a linear change in utility over time. Imbalances in baseline values will be adjusted for using multiple regression [52].

Only health care costs assumed to be affected by the PACT model and of potential economic significance will be included in the main analysis. Costs borne by patients and productivity losses to society are considered less relevant for the patient group in this study. Two main cost components will be included: the costs related to the PACT model (technology and personnel costs) and the cost related to health services use over the course of the trial (hospital inpatient, outpatient, day hospital and emergency services, community-based health care services and medication). Cost weights will be applied to calculate the costs associated with each primary and secondary health resource activity. These will be collected from hospital accounting databases, national tariffs and official wage and price lists.

The cost-utility analysis will be based on the net-monetary benefit framework (NMB). The NMB approach allows for modelling the variability in the cost effectiveness between the centres and GPs [53]. NMB regression will be used to analyse whether the PACT model is more cost effective for a specific subgroup of patients (the effect modification in RQ 5).

The data will be analysed using appropriate techniques that account for the correlation between cost and effect data. Trial-wide costs, QALYs and incremental cost-effectiveness ratios will be reported, as will hospital-specific estimates. If the economic evaluation finds that one option ‘dominates’ the other with a negative incremental costs (costs less) and a positive incremental effect (produces more QALYs), this will considered the most cost-effective option. If better outcomes are associated with higher costs, an incremental cost effectiveness ratio (ICER) will be calculated [54]. Cost effectiveness will then be assessed based on different willingness-to-pay (WTP) thresholds.

Sampling uncertainty will be handled through the use of p-values and confidence intervals (CIs) for costs, QALYs and incremental cost-effectiveness ratios. Confidence intervals for the mean differences in costs and effects between groups will be calculated using non-parametric bootstrap simulation. Cost-effect pairs will be plotted in the cost-effectiveness plane and show the 95 % confidence regions for the ratio. A range of different thresholds will be analysed using a cost-effectiveness acceptability curve.

Non-sampling uncertainty will be handled in sensitivity analyses. The most uncertain methodological assumptions will be varied to illustrate the robustness of the results. Sensitivity analyses will be performed on perspective (societal versus health provider) and different willingness-to-pay values.

#### Model-based economic evaluation

Decision modelling will be used to address cost effectiveness beyond the 12-month study period. A Markov model will be developed to calculate the costs and QALYs with a 10-year time horizon. The model will combine the results from the trail-based analysis with data from the literature and official Norwegian statistics. Future costs and QALYs will be discounted according to Norwegian guidelines for health economic evaluations [55]. Probabilistic sensitivity analysis will be conducted to test parameter uncertainty and construct cost-effectiveness acceptability curves.

### Ethics

The study has been granted ethical approval by the Regional Committees for Medical and Health Research Ethics in Norway (REK No. 2014/1707). We have been granted approval to use electronic hospital record (EHR) data to identify potential controls so that invitations to participate in the trial can be issued. We have also been granted approval to include patients unable to provide informed consent (due to cognitive impairment) as long as the next of kin provides informed consent on the patient’s behalf. All data will be temporarily stored on a secure research server at UNN. The local data protection supervisor has approved the study. The trial has been registered at ClinicalTrials.gov.

## Discussion

The PACT model is now being established at two hospital in the northernmost region in Norway. It is a comprehensive integrated care model that aims to ensure the safe discharge and prevent hospital admissions of frail elderly patients with multiple conditions. As part of the PACT intervention, a research project evaluating the effect on patients’ health and functional status, health resource use and costs will be carried out. The present paper has described the PACT model and the rationale for and design of the effectiveness and cost-effectiveness study.

The project boasts the committed engagement of leaders and health personnel from all three main health care organisations in the area. The municipal nursing services, the GP-services and the specialist services of the hospitals are collaborating to improve service integration. The implementation of patient-centred teams is financed as a cost-sharing collaborative, where all three health organisations fund the project leader, the hospitals and nursing services fund the personnel resources used by the team, and the research project is funded by a three-year research grant from the regional health authorities.

Complex interventions have multiple, interacting and context-dependent active ingredients, which are challenging to identify and keep stable [56]. In this project, the CCM, with its theoretical rationale, has inspired the design of the new service model. However, the PACT model is complex in the sense that each of the ingredients—those of the team and the approach to the patient—is tailored to the patient and the circumstances at every point in time. The active ingredient in this picture cannot be isolated to one single thing. The factors act synergistically to create a new and sustained practice. The most stable component is the theoretical concept, in this case, the CCM, which includes a description of structures, roles and supporting elements. This means that fidelity to the theoretical background is an important measure. In our case, we measure fidelity through the PACIC questionnaire, which evaluates the chronic care experiences of patients. However, the final decisions lie with health care management and not with the researchers. We accommodate this in our design through a close follow-up of the project, which monitors the changes and allows sub-analyses to look for effects that are in line with major changes in the project.

Dedicated, interdisciplinary patient-centred teams can be a new, more effective and cost-effective way to provide health care to older patients with long-term complex needs. Recent studies and reviews underpin the importance of strategies that support patient involvement, engagement and self-care [57–59]. The active two-way patient–provider dialogues and practical skill development in self-management are reported to be important for better outcomes [60]. The patient-centred teams will ensure that several elements of self-management, such as goal setting, self-monitoring and care plans, will be realised, thereby providing more insights into the relevance of self-management in terms of outcomes and cost effectiveness. In the United Kingdom, the transformation from conventional to patient-centred care in the health system has been estimated to have a cost-cutting potential of 7 % [61].

The strengths of the present study are its real world setting and the involvement of designated health professionals in designing and developing the integrated care model. The PACT model has been initiated and is solely driven by managers and clinical personnel at the hospital and the community services. The structure and organisation of the team and how they work will be continuously adapted to the clinical and everyday routines at the hospitals and in the home care settings. This might increase the probability of sustaining the PACT model after the project has ended.

The main limitation of the study is the lack of randomisation. Although we recognise that randomisation is the most robust method of avoiding systematic bias between comparison groups, this method is not possible in this trial. The PACT model aims to improve geriatric care in terms of both structures and routines at the organisational level of two hospitals. A patient-level randomisation, which requires the organisation to switch between old “usual care” and a new team-based proactive care, is unlikely to be successful [56]. Randomising the organisations could be an alternative, but with only four hospitals, this is too small to make cluster randomisation meaningful. We are therefore using a matched control group with before-and-after comparisons. This design is approved by Cochrane for use in systematic reviews of interventions “*that cannot be randomized, or which are extremely unlikely to be studied in randomized trials*” [28].

This evaluation study contributes to the research literature by establishing the effectiveness and cost effectiveness of proactive, patient-centred team work. Research on how the PACT model will affect health outcomes, functional status and health care costs can be useful information for decision makers in the field. Based on this research, the local health authorities can make informed decisions about whether to continue to support and fund the PACT model. Furthermore, the results can be used to determine whether the PACT model should be expanded to other hospitals and municipalities in the region. This research can also be used as the basis for implementing patient-centred interdisciplinary teams in other health regions, both nationally and internationally.
